# Deep Learning Approach for Multimodal Biometric Recognition System Based on Fusion of Iris, Face, and Finger Vein Traits

**DOI:** 10.3390/s20195523

**Published:** 2020-09-27

**Authors:** Nada Alay, Heyam H. Al-Baity

**Affiliations:** 1 Tabadul Company, Riyadh 11311, Saudi Arabia; noalay@tabadul.sa; 2 Information Technology Department, College of Computer and Information Sciences, King Saud University, Riyadh 11362, Saudi Arabia

**Keywords:** deep learning, convolutional neural network, biometric systems, multimodal biometric system, biometric fusion, iris recognition, face recognition, finger vein recognition, recognition system

## Abstract

With the increasing demand for information security and security regulations all over the world, biometric recognition technology has been widely used in our everyday life. In this regard, multimodal biometrics technology has gained interest and became popular due to its ability to overcome a number of significant limitations of unimodal biometric systems. In this paper, a new multimodal biometric human identification system is proposed, which is based on a deep learning algorithm for recognizing humans using biometric modalities of iris, face, and finger vein. The structure of the system is based on convolutional neural networks (CNNs) which extract features and classify images by softmax classifier. To develop the system, three CNN models were combined; one for iris, one for face, and one for finger vein. In order to build the CNN model, the famous pertained model VGG-16 was used, the Adam optimization method was applied and categorical cross-entropy was used as a loss function. Some techniques to avoid overfitting were applied, such as image augmentation and dropout techniques. For fusing the CNN models, different fusion approaches were employed to explore the influence of fusion approaches on recognition performance, therefore, feature and score level fusion approaches were applied. The performance of the proposed system was empirically evaluated by conducting several experiments on the SDUMLA-HMT dataset, which is a multimodal biometrics dataset. The obtained results demonstrated that using three biometric traits in biometric identification systems obtained better results than using two or one biometric traits. The results also showed that our approach comfortably outperformed other state-of-the-art methods by achieving an accuracy of 99.39%, with a feature level fusion approach and an accuracy of 100% with different methods of score level fusion.

## 1. Introduction

The acceleration of the emergence of modern technological resources in recent years has given rise to a need for accurate user recognition systems in order to restrict access to the technologies. The biometric recognition systems are the most powerful option to date. Biometrics is the science of establishing the identity of a person through semi- or fully-automated techniques based on behavioral traits, such as voice or signature, and/or physical traits, such as the iris and the fingerprint [1]. The unique nature of biometrical data gives it many advantages over traditional recognition methods, such as passwords, as it cannot be lost, stolen, or replicated [2]. Biometric traits can be categorized into two groups: extrinsic biometric traits such as iris and fingerprint, and intrinsic biometric traits such as palm vein and finger vein. Extrinsic traits are visible and can be affected by external factors, while the intrinsic features cannot be affected by external factors [3].

In general, the biometric recognition system consists of four modules: sensor, feature extraction, matching, and decision-making modules [1]. There are two types of biometric recognition systems, unimodal and multimodal. The unimodal system uses a single biometric trait to recognize the user. While unimodal systems are trustworthy and have proven superior to previously used traditional methods, but they have limitations. These include problems with noise in the sensed data, non-universality problems, vulnerability to spoofing attacks, intra-class, and inter-class similarity [4].

Basically, multimodal biometric systems require more than one trait to recognize users [1]. They have been widely applied in real-world applications due to their ability to overcome the problems encountered by unimodal biometric systems [4]. In multimodal biometric systems, the different traits can be fused using the available information in one of the biometric system’s modules. The four types of fusion include sensor-level fusion, feature-level fusion, score-level fusion, and decision-level fusion. The advantages of multimodal biometric systems over unimodal systems have made them a very attractive secure recognition method [1].

Several biometrics researchers have relied on machine learning algorithms for recognition purposes [5,6,7,8,9]. Machine learning algorithms require some extraction techniques to extract features from raw biometric data and transform the raw data into an appropriate format before classifying it. Moreover, machine learning algorithms require some preprocessing steps to be carried out prior to feature extraction. Furthermore, some extraction technologies do not always work successfully with different types of biometrics or different data sets of the same biometrics. Additionally, they cannot cope with biometric image transformations, for example, zooming and rotation [10].

Recently, deep learning has made a considerable impact and produced excellent results in biometrics systems [11,12,13,14,15,16,17,18,19,20]. The deep learning algorithms have overcome many of the limitations of other machine learning algorithms, particularly those associated with feature extraction techniques. Deep learning algorithms can cope with biometric image transformations and can extract features from raw data [21].

In view of the great performance of deep learning methods in various recognition tasks, this study aims to intensively investigate the use of the convolutional neural network (CNN) algorithm in recognizing a person from three biometric traits, which are face, iris, and finger vein. In this paper, an efficient multimodal biometric human identification system is proposed based on building a deep learning model for images of the face, iris, and finger vein of a person. These traits have been chosen as the face is perhaps the most natural and, therefore, obvious individual recognition trait, while the unique and highly precise nature of recognition information contained in the iris makes it an effective option. The third trait, finger vein, has been added in regard to enhancing the accuracy of the identification result and improving the security and reliability of the proposed model. Finger vein is an intrinsic biometric trait, and unlike a fingerprint, finger vein cannot be affected by external factors and cannot be easily damaged, forged, or obscured. Moreover, the finger vein provides clear-cut dissimilarities between persons. At present, research on the fusion of these three types of biometrics is still very limited. To the best of our knowledge, no work has been carried out on a multimodal identification biometric system using three traits, one of which is finger vein. In addition, the paper explores fusing the traits at two fusion levels: feature level fusion and score level fusion using two score methods, namely, the arithmetic mean rule and the product rule. SDUMLA-HMT, a publicly available real multimodal biometric dataset, is used for system evaluation. The proposed identification system relies on end-to-end CNNs models that extract features and then classify the person without deploying any image segmentation or detection techniques.

The paper is organized as follows: Section 2 presents an overview of existing research on multimodal biometric systems; Section 3 presents a description of the methodology; Section 4 provides a detailed description of experimental results. Section 5 discusses and analyzes the results. Section 6 concludes the paper and discusses potential future work.

## 2. Related Work

Several studies have proposed multimodal biometric systems that utilized a variety of recognition techniques. This section contains a review of recent studies that employed traditional machine learning and deep learning approaches in multimodal biometric systems.

Discovering ways to combine different physical biometric traits has underpinned several recent biometric recognition studies. Bouzouina and Hamami [5] proposed a multimodal verification system that fused the face and iris traits at feature level fusion. The research employed various methods of feature extraction and applied support vector machines (SVM) algorithm for user verification and it produced an accuracy of 98.8%. Hezil and Boukrouche [6] proposed a biometric system that used the ear and palm print traits and fused them at the feature level. They developed texture descriptors and three classification methods. In another study, Veluchamy and Karlmarx [7] used unusual physical traits, finger vein, and knuckle traits to develop a multimodal biometric identification system. The system fused the traits at the feature level. The authors employed the K-SVM algorithm and their system achieved an accuracy of 96%.

Recently, Chanukya et al. [8] used the neural network to build a multimodal biometric verification system that recognized a human from their fingerprint and ear images. The system developed the modified region growing algorithm to extract shape features from the traits, and local Gabor Xor pattern to extract texture features from the traits. The proposed system achieved an accuracy of 97.33%. Furthermore, Ammour et al. [9] proposed a new feature extraction technique for a multimodal biometric system that relayed on face and iris traits. The iris feature extraction was carried out with a multi-resolution 2D Log-Gabor filter. While the facial features were extracted using singular spectrum analysis and normal inverse Gaussian. For the classification, fuzzy k-nearest neighbor (K-NN) was employed. The feature fusion was performed using score fusion and decision fusion.

On the other hand, some studies have focused on recognizing the users by behavioral biometric traits. In these systems, the feature recognition and extraction are difficult since behavioral traits do not offer reliably repeated patterns. Panasiuk et al. [11] tackled this problem by developing a system using K-NN classifier that recognized the user from a combination of mouse movement and keystroke dynamics. The proposed system reached an accuracy rate of 68.8%.

One of the studies that used deep learning algorithms for building a biometric system was conducted by Ding et al. [12]. In this study, a deep learning framework for face recognition was proposed. The framework used multiple face images and comprised eight CNNs for feature extraction and a three-layer stacked auto-encoder (SAE) for feature level fusion. Two different datasets were used to train the CNNs, namely CASIA-WebFace and LFW, which achieved accuracy rates of 99% and 76.53%, respectively. A study conducted by Al-Waisy et al. [13] proposed a multimodal biometric system for user identification, called IrisConvNet, which combined both the right and left irises using ranking-level fusion. The system firstly detected the iris region in the eye image, and then this detected region was entered into the CNN model. The system achieved a 100% recognition rate. In another study by the same authors, Al-Waisy et al. [14] developed a biometric identification system based on the face, and left and right irises. For face identification, a face detection region method was used, and then a deep belief network (DBN) was applied. For iris identification, IrisConvNet [13] was used. Various matching score fusion methods were employed and the accuracy of the proposed system was 100%.

Soleymani et al. [15] proposed a multimodal CNN where the iris, face, and fingerprint features were fused at different levels of CNN. The authors used two fusion methods, which were multi-abstract fusion and weighted feature fusion algorithms. The evaluation results showed that using the three types of biometrics gave the best result. In further research [16], the authors proposed a compact bilinear feature fusion algorithm to fuse the features at the fully connected layer. The proposed algorithm was evaluated using different biometric traits (irises, faces, fingerprints) from different datasets. In another study, Gunasekaran et al. [17] proposed a deep contourlet derivative weighted rank (DCDWR) framework. DCDWR used iris, face, and fingerprint traits to recognize a person. Contourlet Transform was applied in the framework for performing preprocessing on input images. A local derivative ternary algorithm was developed for extracting multimodal features. The acquired features from local derivative ternary were fused by using weighted rank level fusion and stored in a database for matching. Finally, a deep learning template matching algorithm was developed to verify the user identity.

Some deep learning research has intended to use finger vein for user recognition. For example, Kim et al. [18] built a multimodal biometric recognition system based on CNN by fusing finger vein and finger shape. A ResNet pretrained model was used, and the two traits fused at the score level using several fusion methods, which were weighted sum, weighted product, Bayesian rule, and perceptron rule. Moreover, Liu et al. [19] used the CNN algorithm to build a recognition system that received multiple finger vein images. They developed the CNN model based on the AlexNet pertained model. The model was trained using the SDUMLA dataset, and it obtained an accuracy rate of 99.53%. Recently, Boucherit et al. [20] used finger vein traits to build an identification model, developing a merge CNN model that used several identical CNNs with different input image qualities. The authors conducted different experiments using different network parameters and layers. The most optimal model took a combination of original images and images enhanced with the contrast limited adaptive histogram (CLAH) method. The model was trained using the FV-USM, SDUMLA-HMT, and THU-FVFDT2 datasets, and it achieved a recognition rate of 96.75%, 99.48%, and 99.56%, respectively.

In general, deep learning models are not trained from scratch; as deep learning demands a large dataset. Training from scratch with deep learning is a lengthy process that involves complex experimentations with different parameter values, for example, weights, number of filters and layers, amongst others. This is the reason why most researchers use pre-trained models, for example, Inception, VGGNet, AlexNet, DenseNet [22]. A previous study by Nguyen et al. [23] proved the success of using five different CNN pre-trained models for iris recognition. The five pre-trained models are VGGNet, Inception, AlexNet, ResNet, and DenseNet. DenseNet achieved the highest accuracy, followed by ResNet, Inception, VGGNet, and AlexNet.

From the previous work in literature, it can be noticed that the most used machine learning algorithm in recent research is SVM [5,6,7]. Moreover, when using machine learning approach to recognize biometrics, the biometrics images require specialized feature extraction algorithms depending on the biometric type. Sometimes the images need several pre-processing stages [5,6,7]. While in deep learning approach, the deep learning network itself extracts the features from the images automatically. The performance of deep learning approaches is generally better than the performance of the machine learning approaches. However, these deep learning-based approaches, while effective, are very computationally expensive and time consuming [11,12,13,14,15,16,17,18,19,20]. In addition, studies deploying deep learning algorithms in multimodal biometric systems [13,14,15,16,17,18,19,20] begin the experimentation process by applying region detection methods prior to entering data into deep learning model. The use of region detection methods requires first selecting a suitable technique for a particular trait, also, the process can be time-consuming [1]. It is also noticed that the physical biometric traits deliver better performance than behavioral biometric traits [11]. Moreover, the iris trait has a tendency to increase the accuracy rate [13,14]. The last important note is that few studies have used finger vein trait in biometric multimodal systems with deep learning models.

Based on what has been observed in previous studies, this work develops an identification multimodal biometric system that combines face, iris, and finger vein images using the CNN model. Feature level fusion and score level fusion, with different score methods, were applied in order to find out the most effective approach. An end-to-end CNN algorithm will be used to extract features and recognize images.

## 3. Proposed Method

**Figure 1:**
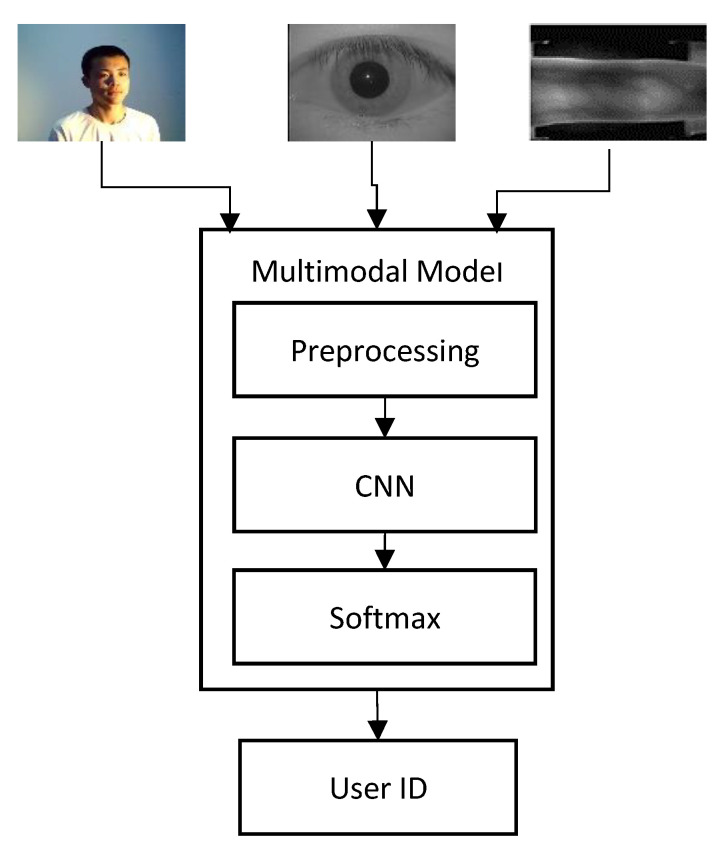
The general structure of the proposed system.

This paper proposes a CNN-based multimodal biometric system using face, iris, and finger vein traits. The general structure of the proposed method is demonstrated in Figure 1. Firstly, the iris, face, and finger vein images of the user are captured. Then, the user identity is recognized by using the multimodal system, which is composed of three fused CNNs for face, iris, and finger vein recognition. Finally, the model produces the user identity.

To build the proposed multimodal biometric system using the three traits (iris, face, and fingerprint), the unimodal iris and face identification models that were built in our previous work [24] were reused in this study. Further, a new finger vein unimodal model has been developed. After that, the proposed multimodal model was developed using the three unimodal models.

In [24], our previous study began with training and testing the model for each trait separately to check the effectiveness of each unimodal model before fusing them to a multimodal model. The CNN algorithm was used for feature extraction and classification. The two traits (face and Iris) were fused using feature-level fusion, which fused the features of the two traits, and score level fusion that fused the similarity scores using the arithmetic mean rule method. In order to train and test the model, three datasets were used, which were the SDUMLA-HMT [25], IT Delhi [26], and FERET [27]. IT Delhi and FERET were used for initial experiments on iris and face unimodal models, respectively. The SDUMLA-HMT dataset was selected to evaluate the performance of the multimodal system. The multimodal biometric system in [24] achieved an accuracy rate of 99.22% using the feature-level fusion approach, and achieved an accuracy rate of 100% using the score-level fusion approach.

In this study, the third biometric trait, finger vein, was added due to the importance of this trait in maintaining high recognition accuracy, and at the same time, enhancing system security as each person has a unique finger vein pattern, and vein patterns provide a good distinction between persons. In addition, as a person grows, the finger vein pattern will not change, i.e., vein patterns are permanent and do not change with time. Finger veins, unlike face or fingerprint traits, are inside the body and this makes them virtually impossible to replicate [28]. To the best of our knowledge, no work has been conducted on multimodal identification biometric system with three traits, one of which is finger vein trait.

Basically, the model for finger vein recognition was built first, and after that, the three CNNs unimodal models (face, iris, and finger vein) were fused at the feature level and score level by employing two score fusion methods. The feature-level fusion was selected because the fusion is performed before the matching module, and generally, fusion before the matching module is effective since the data have rich information about the features [29]. Score level fusion was chosen because of the strong trade-off between the simplicity in merging the traits’ data and better information content. Besides, it is a relatively straightforward approach for combining scores generated by the different CNNs models [29].

**Figure 2:**
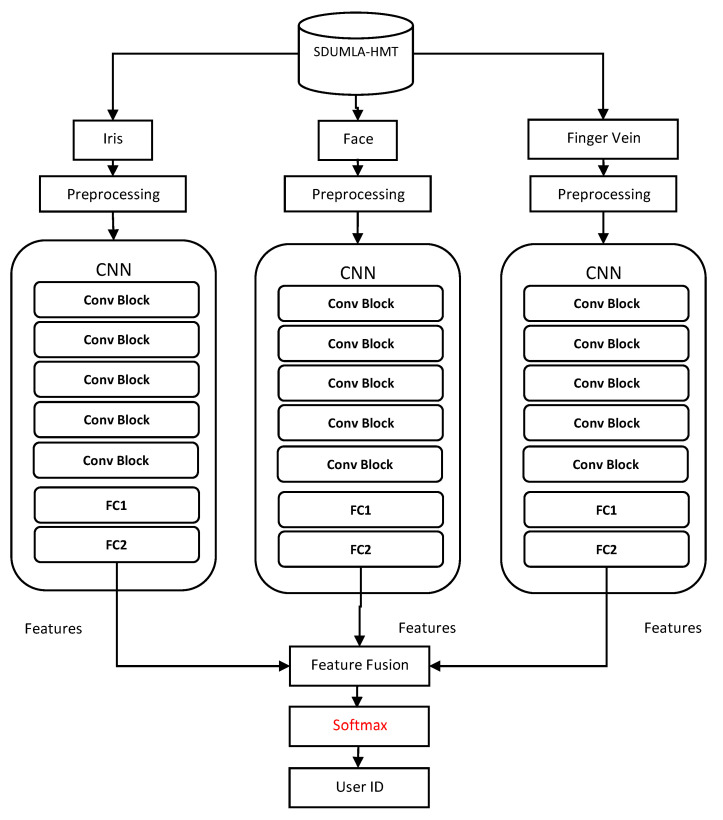
The framework of the multimodal biometric model using feature-level fusion approach. Conv Block represents a block of convolutional layers and pooling layers, and FC represents a fully connected layer.

**Figure 3:**
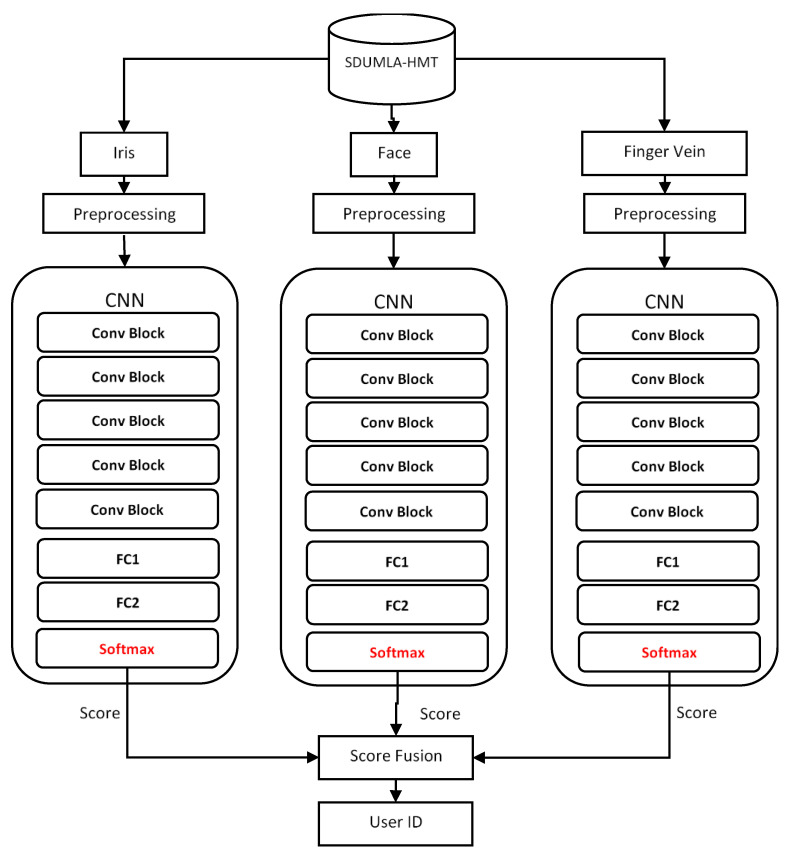
The framework of the multimodal biometric model using a score-level fusion approach. Conv Block represents a block of convolutional layers and pooling layers, and FC is a fully connected layer.

The framework of the proposed multimodal CNN model in this study is demonstrated in Figure 2, which shows the feature level fusion approach, while Figure 3 displays the score level fusion approach. The Figures show that face, iris, and finger vein images are first retrieved from the dataset. Then, some preprocessing actions are performed on the images, such as image resizing and data augmentation. Afterward, each of the biometric traits is fed into its CCN model, and then, the three CNNs are fused. In Figure 2, the fusing is performed at feature level fusion, therefore, the features are combined before the softmax classifier. While in Figure 3, the fusing is done at the score level fusion; thus, the scores are combined after the softmax classifier. Finally, the fused model outputs the user identity. The different parts of the model are described in the following subsections.

### 3.1. CNN Models 

One of the best known and widely used deep learning algorithms in image classification applications is the convolutional neural network (CNN). The CNN architecture comprises three different kinds of layers, known as convolutional, pooling, and fully connected layers. Basically, the CNN algorithm receives an input image, which passes through CNN layers in order to identify its features and recognize it, and then produces the result of classification.

The convolutional layers extract features from images using kernels or filters, which move over the input image to detect information from the image. Filters within the CNN’s first layers identify simple patterns and colors in the image, with more complex patterns and colors detected as the image passes through successive layers. Filters locate features through a convolution process that finally produces a feature map as its output. As the image passes through the pooling layer, the complexity of CNN’s is reduced. The fully connected layers combine features in a one-dimensional vector and give the classification result using the softmax classifier [30].

It is necessary to specify the loss function and optimizer (optimization method) when preparing the CNN algorithm for training; the loss function measures the differentiation between the predicted and actual values. The optimizer is a mathematical function employed in finding values of parameters that minimize the loss of value, for example, the weights matrices [31].

When training the CNN model, two kinds of propagation are used, known as forward propagation and backpropagation. Forward propagation involves the model taking image input and setting filters and other parameter values on a random basis. The input is then propagated forward through the model and uses the random parameters to calculate the loss value. Following this, backpropagation enables the model to use an optimization method to reduce the output loss value. During backpropagation, the loss value of the forward propagation is used to enable model weights and parameters to be modified, and loss value to be reduced accordingly. This prepares the parameters for the next round of forward propagation [22].

Tuning the hyperparameters is an inherent challenge in training a CNN model. Hyperparameter tuning involves identifying the optimum hyperparameter values for the algorithm, as hyperparameters contain all the training variables pertaining to the structure of the model or training algorithms. CNN hyperparameters encompass learning rate value, the number of epochs, dropout layers, L1 and L2 regularization, batch normalization layers, and batch size [22].

**Figure 4:**
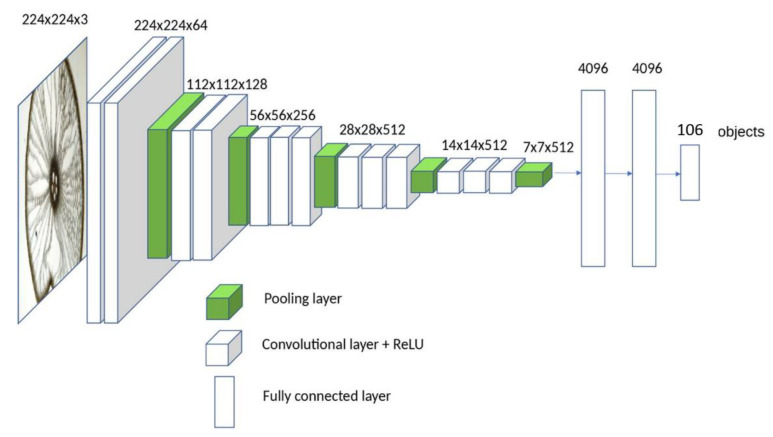
VGG-16 [33].

In our study, VGG-16 [32] as a pre-trained model to identify iris, face, and finger vein was selected since it has a simple CNN architecture, and it is the most widely used in deep learning research to date [22]. VGG-16 receives an input of 224 × 224 × 3. VGG-16 contains 13 convolutional layers, 5 pooling layers, and 3 fully connected layers, as shown in Figure 4. The first convolutional layer uses 64 filters of size 3 × 3, and the size of the resulted feature map is 224 × 224 × 64. VGG-16 uses Rectified Linear Unit (ReLU), which is a non-linear activation function that transfers the output of the convolutional layer to a non-linear output. ReLU replaces negative values with zero, and it is defined as:

y = max (0, x),(1)

where x is the output of the convolutional layer.

In VGG-16, all convolutional layers maintain the same feature map size by using a filter size of 3 × 3 and padding of 1, and only the number of filters changes to 64, 128, 256, and 512. In the pooling layers, max pooling is used. Each pooling layer uses a filter of size 2 × 2, and a number of strides 2 × 2 to reduce the size of the feature map. After the last pooling layer, a feature map of 7 × 7 × 512 pixels is obtained and then passed to the fully connected layer as one vector. The first two fully connected layers have 4096 nodes, and the third fully connected layer used the softmax function for classification, and the layer has 1000 nodes, which represents the 1000 classes in ImageNet [32]. In our model, it was necessary to modify the third fully connected layer with a new one that matches the number of classes in the used dataset, which is 106.

Softmax classifier is a multi-class classification function. It takes a vector of n real numbers where n is equal to the number of classes, and then it normalizes the input into a vector of values that follows a probability distribution whose total sums up to 1. The output values are between 0 and 1, which accommodate as many classes in the neural network model. Softmax classifier calculates the probabilities of each class over all possible classes, and the class that has the highest probability is the target class. Softmax classifier applies the exponential function to each element in the vector, and then normalizes these values by dividing by the sum of all the exponentials as the following formula:

(2) $$\sigma \left(x_{j}\right)=\;\frac{e^{x_{j}}}{\sum_{i}e^{x_{i}}}$$

where x_j_ is an element in x input vector, and j is the jth class.

The outputted score vector from the softmax classifier can be represented as:

Softmax output = {p1, p2, p3, …, pn},(3)

where the pi is the probability of belonging the data sample to the i class [34].

To build our CNNs models using VGG-16, the first four blocks in VGG-16 weights were frozen, as the base layers’ filters search for low-level features, such as angles and lines, within the images. The top layers, or fifth block, were only trained, where the filters search for high-level features.

### 3.2. Data Pre-Processing

Two preprocessing techniques were applied: image resizing and data augmentation. To make the images suitable for use with the VGG-16 model, images were resized to 224 × 224 pixels, and data augmentation was employed to increase training data and to reduce overfitting problems. The augmentation techniques that were used to increase the number of iris and finger vein images were rotation, shearing, zooming, width shifting, and height shifting. And to increase the faces images rotation, shearing, zooming, width shifting, height shifting, and horizontal flipping were used.

### 3.3. Fusion Approaches

#### 3.3.1. Feature-Level Fusion

Fusion at the feature level includes the integration of features corresponding to multiple traits. The extracted features from the three traits were fused to create new features that represent the user. In this fusion approach, the model learned how to recognize the combined features during the training phase. The output of the second fully connected layers of the face, iris and finger vein CNNs models are fused. As such, the features vectors that resulted from the second fully connected layer of the three CNN models become one vector, which can be defined as:

(4) $${X\;=\;x}_{r}\left|{\;x}_{f}\right|x_{v}$$

where x_r_ is the extracted features from the iris image, x_f_ is the extracted features from the face image, and x_v_ is the extracted features from the finger vein images.

Then, the resulted vector (X) is entered into the softmax classifier, which classifies the image based on the similarity score and then recognizes the person’s identity.

#### 3.3.2. Score-Level Fusion

In the score level fusion approach, the output of the second fully connected layer of each CNN model for iris, face, and finger vein is entered into its softmax classifier to get the similarity score. The score level fusion approach has two steps. The first step was normalizing the score resulted from each CNN model, and then the scores of the CNNs were fused using a score fusion method. Finally, the model outputs the identity of the person whose fused score value is the highest.

Two different score fusion methods, namely, the arithmetic mean rule and the product rule fusion were employed. The arithmetic mean rule adds the scores for each individual trait, divides the product by the number of traits, thus giving a combined score [35].

The arithmetic mean rule is calculated by the following equation:

(5) $$S\;=\;\sum_{t=1}^{j}S_{t}/j\;,$$

where S_t_ is the score vector of the trait t, and j is the number of traits.

In the product rule, the fused score is calculated by multiplying the scores of the three traits. It calculated as [35]:

(6) $$S\;=\;\prod_{t=1}^{j}S_{t}\;,$$

where S_t_ is the score vector of the trait t, and j is the number of traits.

## 4. Experiments and Results

In this section, the experimental setup and the conducted experiments are described, then the results are set out.

### 4.1. Experimental Setup

The system is implemented using the Google Colaboratory platform (Colab), which is a machine learning research tool that permits users to run the code in a hosted CPU or GPU [36]. Keras Python library [37] is used for developing the proposed model.

This study employed two datasets; FV-USM [38] and SDUMLA-HMT datasets [25]. As a first step of building the proposed multimodal system, the FV-USM dataset is used firstly for training and testing the finger vein unimodal identification model. After that, SDUMLA-HMT is used for evaluating the performance of the proposed multimodal system.

The FV-USM dataset is created by Universiti Sains Malaysia. FV-USM contains finger vein images that were collected from 123 subjects for 83 males and 40 females with age between 20 and 52. The dataset has different 2952 finger vein images that were captured from the 123 subjects [38]. In our study, the FV-USM dataset was divided into 60:20:20, 60% for the training, 20% for validation, and 20% for testing.

The SDUMLA-HMT dataset is a multimodal biometrics dataset and it is created by a group of machine learning and applications in Shandong university. SDUMLA-HMT stores a range of biometric data gathered from 106 people, including the face, iris, finger vein, fingerprint, and gait. SDUMLA-HMT contains images for 61 males and 45 females aged between 17 and 31. The dataset consists of different images of the five biometric traits for each subject. SDUMLA-HMT has 1060 iris images that were taken from the 106 subjects. The iris images were collected by an intelligent iris capture device that was developed by the University of Science and Technology of China. In the capturing process, the distance between the eye and the device was within 6 cm to 32 cm. As for face images, SDUMLA-HMT includes 8904 different images with variant poses, facial expressions, illuminations, and accessories for 106 subjects. Lastly, SDUMLA-HMT has 3816 finger vein images that were captured by a device that was developed by the Joint Lab for Intelligent Computing and Intelligent Systems of Wuhan University. In the capturing process, different images of the index finger, middle finger, and ring finger of both hands were collected form each subject [25].

In our previous research [24], the images of each subject (class) in SDUMLA-HMT were divided randomly into training, validation, and testing sets using different percentages (80:10:10), (60:20:20), (90:15:5), and (70:20:10). The best results were obtained when using the percentage of (60:20:20), therefore, in this research, the data of each subject is divided into 60:20:20, 60% for the training, 20% for validation and 20% for testing.

The dataset images were organized into three folders for training, validation, and testing, and each folder contains the samples for each subject. The training set was used for training and fitting the deep learning model by using continued forward and backward passes through it, while the validation set was used to evaluate the final model fit using the forward pass only.

The system evaluation process focused on the correctness of user identification, which can be measured through the accuracy metric. Accuracy can be utilized in evaluating the proposed models and exploring the effects of the different hyperparameters. It can be calculated as the ratio of correctly classified images to the total number of images.

(7) $$\mathrm{Accuracy}\;=\;\frac{\mathrm{Number}\;\mathrm{of}\;\mathrm{correctly}\;\mathrm{classified}\;\mathrm{images}\;}{\mathrm{Total}\;\mathrm{number}\;\mathrm{of}\;\mathrm{images}}\;\times 100$$

### 4.2. Iris Unimodal Experiments

For the iris CNN model, the iris CNN model in our previous research [24] was used. The structure of the CNN model is the same as that of the VGG-16 model, with some modifications that were made to avoid overfitting. After each fully connected layer, a batch normalization layer was added. One dropout layer was added before the classifier, and the dropout rate was set to 0.3. L2 regularization was added to the two fully connected layers and set to 0.00001. the model is trained using a batch size of 32, with a learning rate of 0.00001 and 30 epochs. The Adam optimization function [31] and the categorical cross-entropy loss function were employed. After testing the model using IT Delhi dataset, it produced an accuracy rate of 99.55%, and an accuracy of 98.58% when the SDUMLA-HM dataset was used.

### 4.3. Face Unimodal Experiments

For the face CNN model, the face CNN model in our previous research [24] was used. The model is similar to VGG-16, with some additional layers. After each convolutional layer in the fifth block of the model, a batch normalization layer was added. A batch normalization layer was also added after each fully connected layer. Additionally, a dropout layer before the classifier with the rate set as 0.3 was added. L2 with a value of 0.00001 proving the best, since it produced a lower loss value. The employed learning rate value was set to 0.00001, with a selected batch size of 32 and 45 epochs. The Adam optimization function [31] and the categorical cross-entropy loss function were employed. The results of the face model using the FERET dataset attained an accuracy rate of 98.90%, and an accuracy of 98.72% when the SDUMLA-HMT dataset is used.

### 4.4. Finger Vein Unimodal Experiments

In order to identify the optimum model design for finger vein recognition, many experiments on the finger vein model were conducted in this study. Experiments were carried out during the training phase using learning rate values between 0.00001 to 0.001. Moreover, the model was trained using batch sizes of 32 and 64. Different epoch numbers ranging from 25 to 50 were also tested. The dropout rate was set to values between 20% and 40%.

The structure of the finger vein CNN model is the same as that of the VGG-16 model with some modifications that were applied in order to avoid the overfitting issue. A batch normalization layer after each fully connected layer was added. L2 regularization was added to the two fully connected layers and set to 0.00001. One dropout layer before the classifier was also added and set to 0.3. The model was trained using a batch size of 32, with a learning rate of 0.00001 and 25 epochs. For validation purposes, firstly, the model was tested using the FV-USM dataset [38], which contains only finger vein information. The model achieved an accuracy rate of 98.79% with FV-USM and an accuracy of 98.38% when the SDUMLA-HMT dataset is used.

### 4.5. Multimodal Model Experiments

The multimodal model was created by fusing together the previous three unimodal models; face, iris, and finger vein. The following subsections illustrate the experiments of the two applied fusion approaches.

#### 4.5.1. Feature-Level Fusion

For training the multimodal model, many factors were considered, including learning rates, batch size, and dropout values. It was found that the best model was obtained after the learning rate parameter is tuned to 0.00001, a batch size of 64 and 20 epochs is selected. The dropout layer, with a rate of 0.3, was added before the classifier. The Adam and categorical cross-entropy methods were employed for optimization and loss function. The proposed multimodal model with feature level approach achieved an accuracy rate of 99.39%.

#### 4.5.2. Score-Level Fusion

The classification scores of iris, face, and finger vein models were combined using two different score fusion methods: the arithmetic mean rule and product rule. The accuracy value of the system when these methods were used was 100%.

## 5. Discussions of Results

The identification accuracy results of the conducted experiments are summarized in Table 1 and Table 2 for the unimodal and multimodal models, respectively. The results demonstrate that higher accuracy rates were obtained by the multimodal biometric model in comparison to those of unimodal models. This shows that, as originally proposed, multimodal biometrics provides a highly effective way to improve the accuracy rates of a biometric system. For instance, the developed finger vein unimodal model obtained an identification accuracy of 98.38%, which was less than the accuracy of the proposed multimodal model (99.39%).

A comparison between the achieved results of the proposed multimodal model with the results of our previous work [24] was made based on the type of fusion approach used, as shown in Table 2. For the feature fusion approach, it is worth noting that the proposed multimodal model using the three biometric traits (accuracy of 99.39%) outperformed the multimodal model of the two traits (accuracy of 99.22%) in [24]. Generally, a higher recognition accuracy was obtained by fusing three traits compared to the performance based only on one or two traits in the decision-making process. For the score fusion approach, the proposed model and our previous model in [24] obtained the same results (accuracy of 100%). It can be noticed that the sum rule and the arithmetic mean methods achieved the same results. This could be explained by the fact that both methods are rule-based score fusion methods, which means that they are fixed and not trained rules.

Moreover, it is to be noted that the score level fusion achieved better identification accuracy than the feature level fusion. This can be related to the softmax classifier; in the feature level fusion, the softmax classifier receives a vector, which contains a combination of different features sets extracted from the multiple biometric traits, and then the classifier produces the final score. While in the score level fusion, there are three softmax classifiers, and each of them received a vector of one trait’s feature to produce the score then these scores were combined using a fixed rule method.

**Figure 5:**
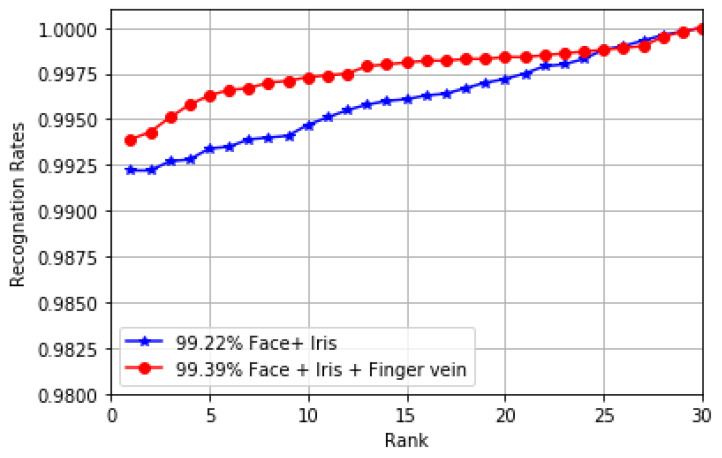
CMC curves for the multimodal models with the feature fusion method.

A performance comparison was carried out between the proposed model and our previous model in [24] based on the feature fusion method, as shown in the cumulative match characteristic (CMC) curve in Figure 5. As can be seen from the figure, the proposed model has achieved better results than our previous model in [24]. Rank-1 identification accuracy greater than 99% has been achieved by our previous model [24], while the proposed model achieved a Rank-1 identification accuracy of 99.39%.

**Figure 6:**
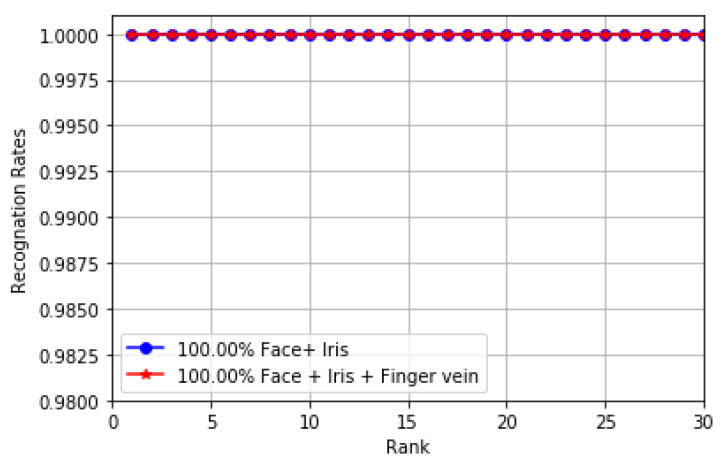
CMC curves for the multimodal models with the score fusion method.

Figure 6 shows the performance of the proposed multimodal model and the performance of our previous model [24] based on the score fusion method. As can be seen, there is not much difference between the performance of the two models when using the score fusion method. It is possible to achieve a rank-1 identification accuracy of 100% using our proposed model and previous model.

For comparative purposes, the developed finger vein unimodal model, as well as the proposed multimodal model, were compared with the other previous studies in the literature. Firstly, the developed finger vein unimodal identification model was compared with other previous models, as shown in Table 3. Table 3 illustrates two previous studies that used the SDUMLA-HMT dataset for building a deep learning model for finger vein identification. The finger vein unimodal model in [20] gave a recognition rate of 99.48%, which has exceeded our model. This result may be attributed to the fact that the model in [20] uses multiple instances of finger vein rather than one instance as we do in our study. The model [20] takes five images of finger vein and this makes the chance for identifying the person higher than using only one finger vein instance. Moreover, the unimodal model in [39] outperforms our finger vein identification model. This is because of the VGG-16 pretrained model that was employed in our finger vein model, which takes a square image and the finger vein images are basically of a rectangular shape in the dataset. This implies the need for resizing the images, which can cause a loss of some information. The CNN model in [39] takes rectangular images to reduce the chances of distortions.

As far as we know, there are no previous studies that have built multimodal biometric models for identifying a person using three traits, one of which is finger vein. Moreover, there is no work in the literature that used the SDUMLA-HMT dataset for evaluating a deep learning model in identifying a person based on the same three biometric traits that are used in our study. Thus, the proposed multimodal biometric model was compared with a previous study [14] that used the SDUMLA-HMT dataset in developing a model for identifying the person based on right iris, left iris, and face traits. Moreover, the proposed multimodal model is compared with the other two previous studies in the literature [15,16], that used three different traits in a multimodal biometric system using different datasets. Table 4 shows the compression results between the proposed model and other previous models.

Although the multimodal biometric system in [14] was able to obtain a 100% identification rate, this is due to the fact that it is applying multiple preprocessing steps on the image to detect specific areas before inserting the image into the deep learning algorithm. This is a time-consuming process, which increases the model execution time. In addition, the accuracy rate of the face identification model, which is based on the DBN algorithm in [14] was 85.34%. However, the multimodal system in [14] relied more on the excellent architecture of the left and right iris CNN algorithms for the fusion process in order to achieve high recognition accuracy. Furthermore, our face identification model accuracy surpassed the accuracy of the face identification model in [14], i.e., the CNN algorithm performed better than the DBN algorithm.

Table 4 also shows the superiority of the proposed model to the other multimodal biometric systems in [15,16]. This distinction in the results of the proposed system was due to an important factor, which was the addition of the finger vein trait, which outperformed the fingerprint in terms of increasing the accuracy in identifying a person. Another reason for this is that the fusion process in [15] was done at different feature level abstraction in the CNN algorithm rather than fusing features at one of the last feature levels, which contains all features of the image. On the other hand, the multimodal model in [16] uses a generalized compact bilinear feature fusion algorithm, which is different than our method of fusing features.

## 6. Conclusions

To conclude, in this paper, a multimodal biometric model was developed for user identification. The proposed system employed the CNN deep learning algorithm. Feature level fusion and two different score fusion methods were deployed to identify the user from their iris, face, and finger vein traits. To our knowledge, this is the first study to investigate the use of deep learning algorithms for a multimodal biometric model with these three traits. Moreover, as mentioned earlier, no work has been conducted on multimodal identification biometric system with three traits, one of which is the finger vein trait. The proposed model used three CNNs to identify each trait. The model performance was evaluated using the SDUMLA-HMT dataset. Generally, the score level fusion approach obtained better accuracy (100%) than the feature level fusion (99.39%). The experimental results showed the excellent performance of the CNN algorithms. It also illustrated that using three biometric traits in identification systems can obtain better results than using two or one biometric traits. The results also revealed that incorporating finger vein trait into a multimodal biometric model could lead to a better identification accuracy when compared with fingerprint trait.

In terms of further research, the authors intend to build CNNs from scratch that are suitable for each trait instead of using a pretrained model. For example, building a CNN for finger vein, which accepts rectangular images. In addition, the authors intend to explore the effect of using deep learning algorithms on a wider range of recognition traits, for instance, DNA, signature, or hand geometry. It will also be interesting to broaden the range of experiments to test the proposed model with different level fusion methods and various multimodal datasets.

## Figures and Tables

**Table 1:** Accuracy results using unimodal biometrics systems.

Biometric Model	Traits	Accuracy
Unimodal Biometric	Iris [24]	98.58%
	Face [24]	98.72%
	Finger vein	98.38%

**Table 2:** Accuracy results using the multimodal biometrics systems.

Biometric Model	Fusion Approach		Accuracy
Previous model of multimodal biometric (face and iris) [24]	Feature level fusion		99.22%
	Score level fusion	(Arithmetic mean rule)	100%
Proposed model of multimodal biometric (face, iris, and finger vein)	Feature level fusion		99.39%
	Score level fusion	(Arithmetic mean rule)	100%
		(Product rule)	100%

**Table 3:** Comparison between the finger vein unimodal model with previous models.

Model	Identification Accuracy
Proposed finger vein unimodal model	98.38%
Boucherit et al. [20]	99.48%
Das et al. [39]	98.90%

**Table 4:** Comparison with previous studies.

	Traits	Level Fusion	Accuracy
Al-Waisy et al. [14]	Right iris, left iris, and face	Score level fusion	100%
Soleymani et al. [15]	Face, iris, and fingerprint	Feature level fusion	99.34%
Soleymani et al. [16]	Face, iris, and fingerprint	Feature level fusion	99.30%
Our proposed model	Face, iris, and finger vein	Feature level fusion	99.39%
		Score level fusion	100%
